# The impact of regionalized trauma care on the distribution of severely injured patients in the Netherlands

**DOI:** 10.1007/s00068-021-01615-1

**Published:** 2021-03-12

**Authors:** Suzan Dijkink, Erik W. van Zwet, Pieta Krijnen, Luke P. H. Leenen, Frank W. Bloemers, Michael J. R. Edwards, Dennis Den Hartog, Peter A. Leenhouts, Martijn Poeze, W. Richard Spanjersberg, Klaus W. Wendt, Ralph J. De Wit, Stefan W. A. M. Van Zuthpen, Inger B. Schipper

**Affiliations:** 1 Department of Trauma Surgery, Leiden University Medical Center, Post zone K6-R, P.O. Box 9600, 2300 RC Leiden, The Netherlands; 2 Department of Medical Statistics and Bioinformatics, Leiden University Medical Center, Leiden, The Netherlands; 3 Department of Surgery, University Medical Center Utrecht, Utrecht, The Netherlands; 4 Department of Surgery, VU University Medical Center, Amsterdam, The Netherlands; 5 Department of Trauma Surgery, Radboud University Medical Center, Nijmegen, The Netherlands; 6 Trauma Research Unit, Department of Surgery, Erasmus MC, University Medical Center Rotterdam, Rotterdam, The Netherlands; 7 Department of Surgery, Academic Medical Center, Amsterdam, The Netherlands; 8 Department of Surgery, Maastricht University Medical Center, Maastricht, The Netherlands; 9 Department of Trauma Surgery, Isala Klinieken, Zwolle, The Netherlands; 10 Department of Trauma Surgery, University Medical Center Groningen, University of Groningen, Groningen, the Netherlands; 11 Department of Trauma Surgery, Medisch Spectrum Twente, Enschede, The Netherlands; 12 Department of Surgery, Elisabeth Tweesteden Ziekenhuis, Tilburg, The Netherlands

**Keywords:** Trauma system, The Netherlands, Regionalization of care, Trauma care

## Abstract

**Background:**

Twenty years ago, an inclusive trauma system was implemented in the Netherlands. The goal of this study was to evaluate the impact of structured trauma care on the concentration of severely injured patients over time.

**Methods:**

All severely injured patients (Injury Severity Score [ISS] ≥ 16) documented in the Dutch Trauma Registry (DTR) in the calendar period 2008–2018 were included for analysis. We compared severely injured patients, with and without severe neurotrauma, directly brought to trauma centers (TC) and non-trauma centers (NTC). The proportion of patients being directly transported to a trauma center was determined, as was the total Abbreviated Injury Score (AIS), and ISS.

**Results:**

The documented number of severely injured patients increased from 2350 in 2008 to 4694 in 2018. During this period, on average, 70% of these patients were directly admitted to a TC (range 63–74%). Patients without severe neurotrauma had a lower chance of being brought to a TC compared to those with severe neurotrauma. Patients directly presented to a TC were more severely injured, reflected by a higher total AIS and ISS, than those directly transported to a NTC.

**Conclusion:**

Since the introduction of a well-organized trauma system in the Netherlands, trauma care has become progressively centralized, with more severely injured patients being directly presented to a TC. However, still 30% of these patients is initially brought to a NTC. Future research should focus on improving pre-hospital triage to facilitate swift transfer of the right patient to the right hospital.

## Background

**Fig. 1:**
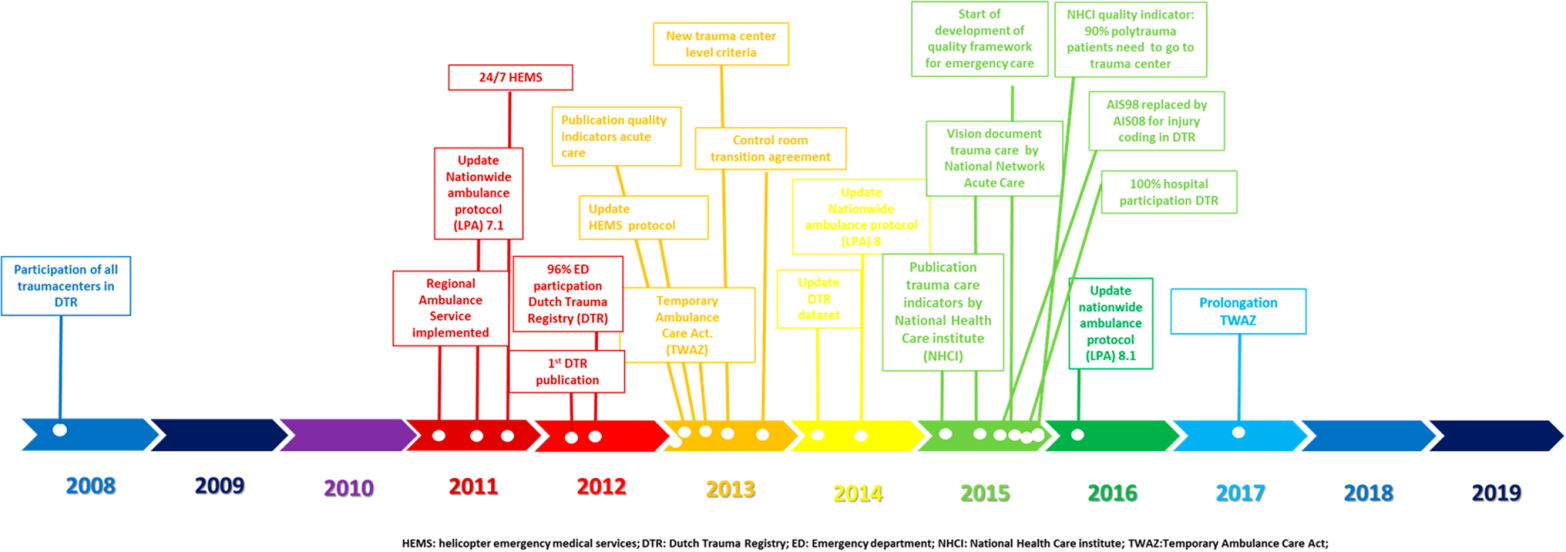
Changes in organization of trauma care in the Netherlands

Following concerns about the organization of both pre-hospital and in-hospital trauma care and the increased public awareness about the importance of well-organized acute care [1, 2], the Dutch government appointed ten trauma centers (TC) in 1998 [3, 4]. Currently, the Dutch trauma care is organized in eleven trauma regions and resembles the American trauma system based on the criteria set by the American College of Surgeons Committee on Trauma [5]. Each region has a catchment area of at least 1.2 million inhabitants with one coordinating TC and several non-trauma centers (NTC) in every region. Since the introduction of the regionalized trauma care, several quality measures were deployed, such as a mandatory participation in the Dutch Trauma Registry [DTR] (2008), which led to 100% participation by all emergency departments in the registry in 2015. Also, level criteria for trauma- and non-trauma centers (2013), and certification for trauma surgeons were implemented. In 2010, ten years after the introduction of well-organized trauma systems, the overall mortality risk after trauma was found to be reduced by 16% [6]. In the past decade, further development of trauma care in the Netherlands concerned, amongst others, the regionalization of the ambulance care including an update of the national guideline for emergency medical service providers (2011), 24/7 availability of helicopter emergency services (HEMS) for acute trauma (2011), yearly quality reports by the Dutch Trauma Registry (2012) and the introduction of trauma-related quality indicators by the Dutch government (2015) (Fig. 1).

Twenty years after the introduction of trauma systems in the Netherlands knowledge about parameters that may influence the distribution of trauma patients is relevant, per se and as a prelude to the analysis of the clinical effects of this concentration of care over time. The objective of this study is to describe the impact of structured trauma care on the distribution of severely injured patients between trauma centers (TC) and non-trauma centers (NTC).

## Materials and methods

### Patients and data

Patients admitted to either one of the appointed regional trauma centers (TCs) or to a non-trauma center (NTCs) are registered in the Dutch Trauma Registry (DTR). This retrospective cohort study included all severely injured patients (Injury Severity Score [ISS] ≥ 16) who were registered in the DTR during the calendar period 2008–2018. Up to 2015, injury coding and calculation of the ISS [7] in the DTR was based on the Abbreviated Injury Scale (AIS) version 1998 [8] and after 2015 on the AIS version 2008 [9]. To enable a comparison of patients’ injury severity over time, a tool developed by Palmer et al. was used to reclassify all AIS1998 injury codes to AIS2008 injury codes [10].

Since 2008, all eleven coordinating TCs contribute data of their admitted trauma patients to the DTR. During the study period (2008–2018), the participation of NTCs in the DTR increased from 62% in 2008 to (near) 100% in 2018 (Table 1). From 2014 on, all Dutch hospitals participate in the DTR.

**Table 1:** Number of trauma centers (TCs) and non-trauma centers (NTCs) participating in the Dutch Trauma Registry (DTR), by calendar year

	**2008**	**2009**	**2010**	**2011**	**2012**	**2013**	**2014**	**2015**	**2016**	**2017**	**2018**
Total number of NTCs with ED	105	104	102	102	101	101	99	96	95	95	91
Number (%) of NTCs participating in the registry	76 (72)	79 (76)	85 (83)	89 (87)	97 (96)	100 (99)	98 (99)	96 (100)	95 (100)	95 (100)	89 (98)
Total number (%) of TCs participating in the registry	11 (100)	11 (100)	11 (100)	11 (100)	11 (100)	11 (100)	11 (100)	11 (100)	11 (100)	11 (100)	11 (100)
Total number of severely injured patients (ISS ≥ 16) in the registry	2350	2450	2479	2968	3394	3578	4006	4205	4422	4450	4694
Number (%) of patients with ISS ≥ 16 and severe neurotrauma	1483 (63)	1515 (62)	1655 (67)	1950 (66)	2211 (65)	2416 (68)	2783 (69)	2413 (57)	241 (55)	2403 (54)	2545 (54)
Number (%) of patients with ISS ≥ 16 without severe neurotrauma	867 (37)	935 (38)	824 (33)	1018 (34)	1183 (35)	1162 (32)	1223 (31)	1792 (43)	2012 (45)	2047 (46)	2149 (45)

*ISS* Injury Severity Score

The distinction between TCs and NTCs is based on the set of trauma center- criteria established by the Dutch Society for Trauma Surgery. TCs (level I centers) in the Netherlands need to have 24/7 multidisciplinary trauma team availability and are equipped for multidisciplinary management of severely injured patients, including the presence of facilities, such as 24/7 angio-interventions, intensive care and specialties like neurosurgery and cardiothoracic surgery. The NTCs are well-equipped trauma-hospitals but lack the 24/7 presence of multidisciplinary trauma teams, and are not appointed primarily to provide care to severely injured patients.

### Statistical analysis

The statistical analyses were performed in R [11] for three types of patients:

A) all severely injured patients (ISS ≥ 16),

B) patients with ISS ≥ 16 and severe (head-AIS ≥ 3) neurotrauma, and

C) patients with ISS ≥ 16 without neurotrauma or with only mild to moderate (AIS ≤ 2) neurotrauma.

Separate analysis of the subgroups with and without severe neurotrauma was considered relevant, since a large part of the patients with ISS ≥ 16 has severe neurotrauma.

**Fig. 2:**
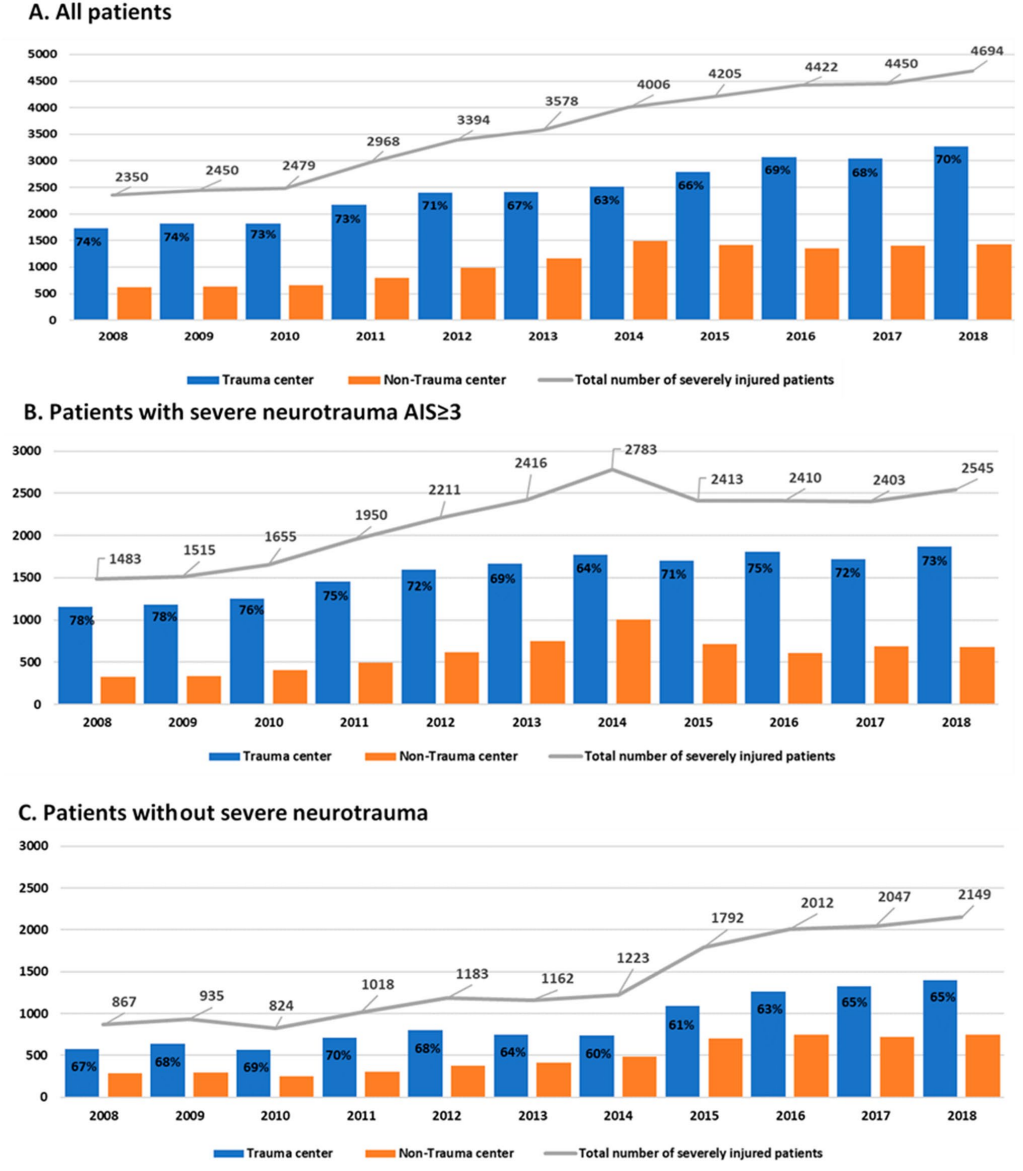
Distribution of severely injured patients (ISS ≥ 16) registered in the Dutch Trauma Registry, directly brought to a level I trauma center or to a non-trauma center over time for **a** all patients, and separately for patients **b** with and **c** without severe neurotrauma, by calendar year

First, the distribution of severely injured patients who were directly brought to a TC and those who were directly brought to a NTC was described over time (Fig. 2).

**Fig. 3:**
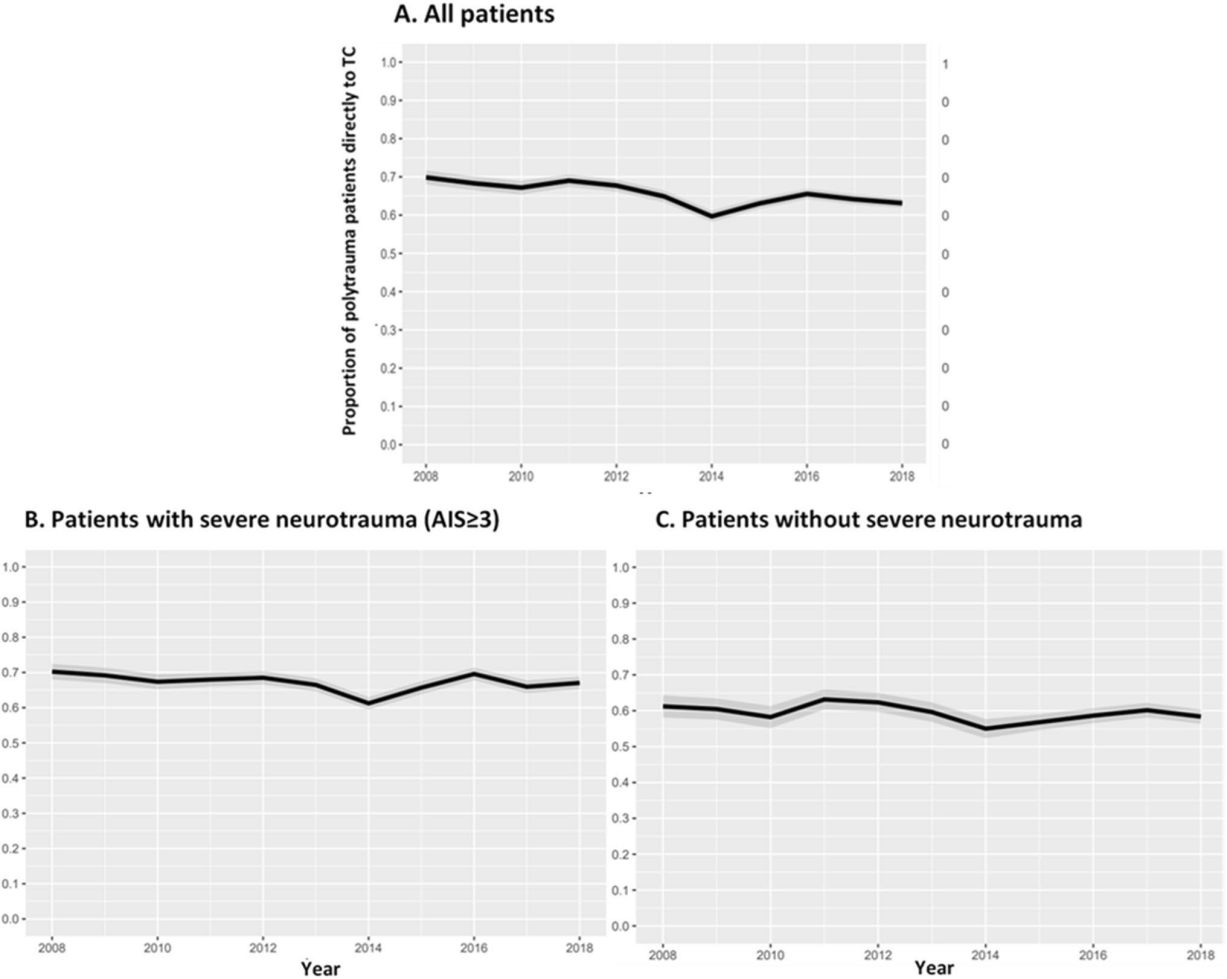
Proportion of severely injured patients (ISS ≥ 16) directly brought to a trauma center, after correction for difference in case mix and for non-participation in the Dutch Trauma Registry, for all patients (**a**), and separately for patients with (**b**) and without (**c**) severe neurotrauma, per calendar year

To assess a potential trend in the proportion of severely injured patients directly brought to a TC over time, the proportion per year was modelled with adjustment for case mix variables (Fig. 3). For this purpose, the PSNL15 case mix model was used, which was developed by the National Network Acute Care (Landelijk Netwerk Acute Zorg, LNAZ), based on the TRISS model [12] and adjusted to the Dutch population [13]. The PSNL15 case mix model includes factors associated with the survival of trauma patients, such as trauma mechanism, vital signs on admission, age and ISS [13]. The proportion of patients that were directly brought to a TC was also corrected for the centers that did not participate in the DTR from the start in 2007. Multiple imputation was used to estimate the number of (severely) injured patients in NTCs for the calendar years in which these centers did not report to the DTR. The multiple imputation for the not reported years was based on the number of and trend in the observed numbers of patients these centers had reported in later years.

**Fig. 4:**
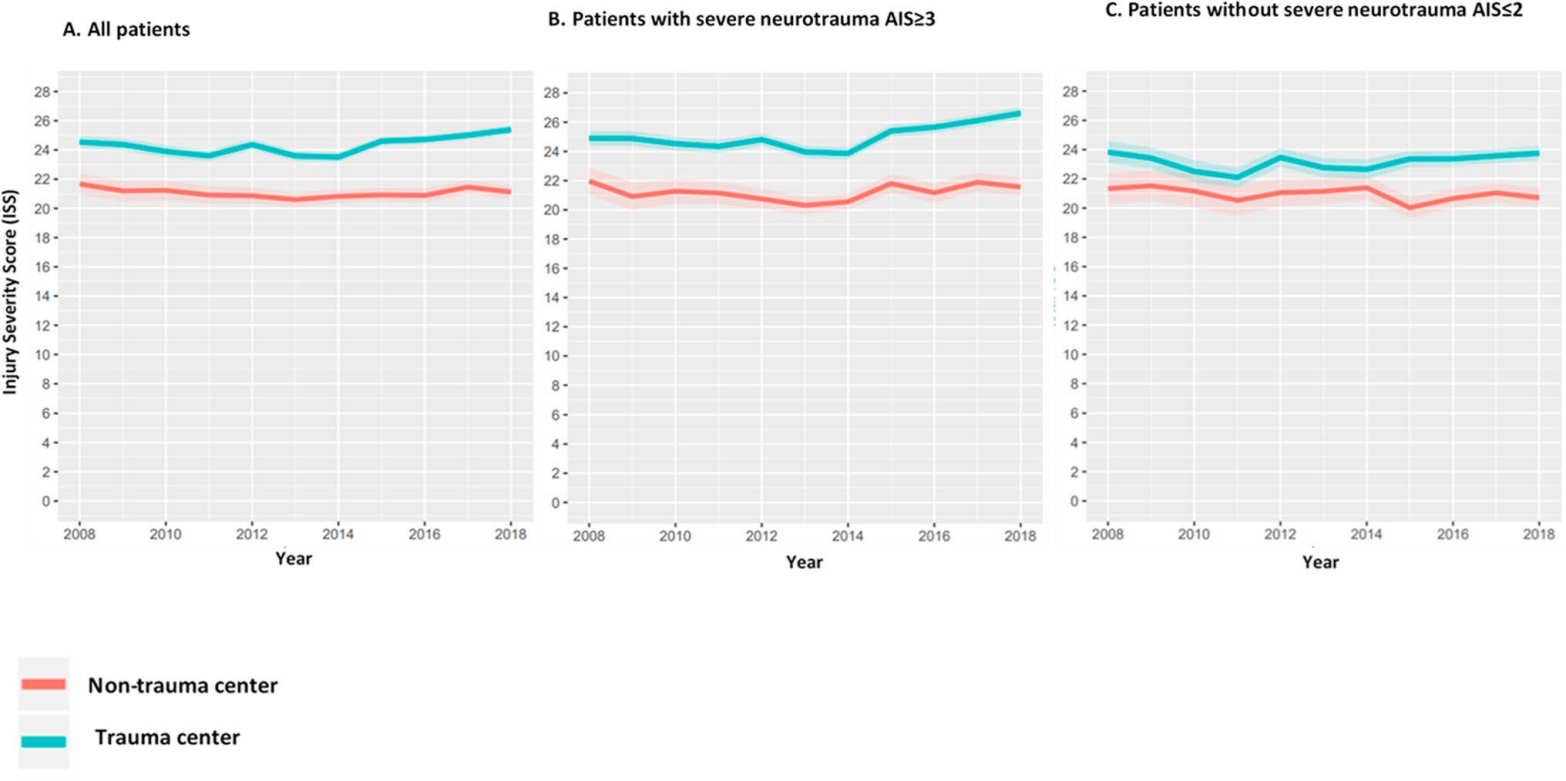
Median Injury Severity Score (ISS) for severely injured patients (ISS ≥ 16) directly brought to a trauma center or a non-trauma center, for **a** all patients, and separately for patients **b** with and (C) without severe neurotrauma, by calendar year

**Fig. 5:**
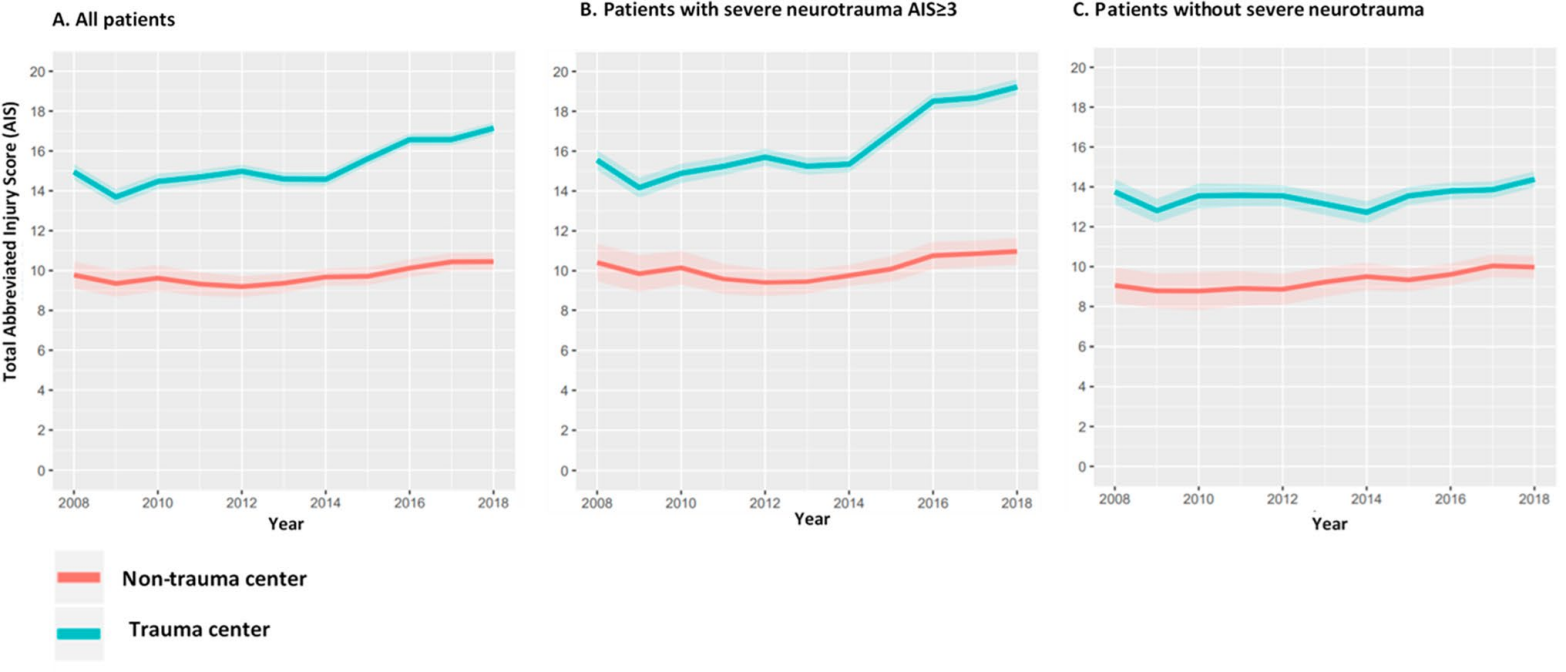
Median total Abbreviated Injury Score (AIS) for severely injured patients (ISS ≥ 16) directly brought to a trauma center or a non-trauma center, for **a** all patients, and separately for patients **b** with and **c** without severe neurotrauma, by calendar year

Second, the median ISS (Fig. 4) and median total AIS (calculated as the sum of all separate AIS severity codes per patient) (Fig. 5) of patients brought to the TCs and NTCs were described over time. The median total AIS was analyzed as several studies have shown that the low interobserver reliability of the ISS limits its use for benchmarking trauma system performance [14, 15]. Total AIS might be a useful marker of injury severity because it includes all injuries (i.e. multiple injuries in one body region) [16].

## Results

### Distribution of severely injured patients

The number of severely injured patients registered in the Dutch Trauma Registry increased from 2350 patients in 2008 to 4694 in 2018 [Fig. 2a]. At the same time, the number of participating NTCs varied from 76 to 100 (72–100%; Table 1). In the years 2008–2014, on average, 66% of all documented severely injured patients had severe neurotrauma, while this was 55% over the years 2015–2018 (Table 1). Both the numbers of registered patients with severe neurotrauma (Fig. 2b) and without severe neurotrauma (Fig. 2c) increased over the 10-year period. The unadjusted proportion of all severely injured patients that were directly brought to a TC was 70% on average. This proportion decreased from 74 to 63% between 2008 and 2014, when the number of NTCs participating in the DTR still increased, and then increased to 70% in 2018 (Fig. 2a). A similar trend was seen in both the subgroup of patients with severe neurotrauma (Fig. 2b) and the subgroup of patients without neurotrauma or with only mild/moderate neurotrauma (Fig. 2c).

A similar trend in the proportion of severely injured patients that were directly brought to a TC was seen after adjustment for variation in case mix and for non-participation of NTCs (Fig. 3). After a decreasing trend between 2008 and 2014, the adjusted proportion increased over the years 2014–2016 and remained stable thereafter. On average, over the past decade, 73% of the patients with severe neurotrauma and 66% of patients without severe neurotrauma were directly transported to a TC.

Similar trends were seen for patients with an ISS > 25 (data not shown), with still 20% of the severely injured patients not primarily transported to a trauma center. Comparable to the ISS ≥ 16 group, patients with severe head trauma (AIS ≥ 3) had a higher chance of being primarily transported to a trauma center than patient without severe head trauma (AIS < 3).

### Injury severity

During the entire study period, the patients directly brought to a TC were more severely injured, reflected by a higher median ISS (22, interquartile range [IQR] 17 27) and higher median total AIS (13, IQR 9–20), than the patients who were directly brought to a NTC (ISS 18, IQR 17- 25 and total AIS 8, IQR 6–12) (Figs. 4 and 5). For all severely injured patients and for the subgroup of patients with severe neurotrauma, the total AIS and ISS in the patients directly brought to a TC (median ISS 24, IQR 17–29 and total AIS 14, IQR 9–21) increased from 2014 onwards (ISS 2014 22, IQR 17–27 vs ISS 2018 25, IQR 19–29 and total AIS 2014 13, IQR 9–20 vs total AIS 2018 17, IQR 12–24), while it remained steady over the years for the patients who were brought to a NTC (ISS 19, IQR 17–25 and total AIS 9, IQR 6–12) (Figs. 4a, b and 5 a, b ). For the subgroup of severely injured patients without severe neurotrauma, the median ISS and total AIS remained similar over time, for both the patients that were directly brought to a TC (ISS 20, IQR 17–25 and total AIS 12, IQR 7–17) and for those directly brought to a NTC ( ISS 18, IQR 17–22 and total AIS 8, IQR 5–12) (Figs. 4c and 5c).

## Discussion

Over the period 2008–2018, the centralization of trauma care in the Netherlands continued. The total number of registered severely injured patients has increased to annually about 4500. This increase is at least partly attributable to increased participation of NTCs in the DTR and to a more accurate registration. As of 2014, all TCs and NTCs with an ED participated in the registry and from then on representative data was available. The proportion of the severely injured patients who were directly brought to a TC slightly increased, and stabilized at 70% in the most recent years. This proportion was somewhat higher for the severely injured patients with severe neurotrauma than for those without neurotrauma or only mild or moderate neurotrauma. The injury severity within the group of severely injured patients that were directly brought to a TC has increased since 2014, especially in the subgroup of patients with severe neurotrauma.

Despite many improvements, challenges remain to be faced. As a consequence of the introduction of a trauma system, severely injured patients are more likely to be admitted to a TC than in the 1990s [6]. However, about 30% of these patients are still transported to NTCs in the Netherlands. Similar percentages are seen in other countries, such as Norway and the United States [17–21]. According to the American College of Surgeons Committee on Trauma, an under-triage rate above 5% is unacceptable, as under-triage increases the risk of mortality and morbidity due to patients not being managed at the best-equipped hospital [22]. In addition, MacKenzie et al. showed in their study that, especially for the younger, more severely injured patients, treatment at a TC is not only more effective but also cost-effective, which underlines the importance of bringing the severely injured to a TC [23]. Studies show that especially the most severely injured patients, with ISS ≥ 25, hemodynamically instable and patients with severe traumatic brain injury (AIS ≥ 3), benefit the most from proper hospital triage, demonstrating lower mortality rates for these patients when brought to a TC [24–27]. Reducing under-triage should therefore be given priority. This does, however, remain a major challenge even in mature trauma systems [22, 28]. Van Rein et al. showed in their systematic review that almost all pre-hospital triage protocols had a low sensitivity and therefore failed to identify all severely injured patients who needed treatment in a TC [29]. Especially identifying serious neurotrauma by EMS providers has proven to be difficult; 32% of all neurotrauma and 21% of the severe neurotrauma are not recognized at the accident scene [30]. Particularly for these patients, the hospital triage may be further optimized by advanced triage tools. In trauma patients, the effects of drugs and alcohol often obscure the real trauma-related neurological symptoms so that symptoms often do not correspond with findings on the CT scans once the patients have arrived at the ED [30].

The current lack of field triage criteria able to adequately predict if a patient will be classified as severely injured contributes to the challenge to fulfill the Dutch Healthcare Institute’s prerequisite of 90% severely injured patients being brought directly to a dedicated TC. In practice, emergency service providers guide their decision whether or not to go to a TC based on their clinical experience, and clinical signs of severe injury at the accident scene in addition to what the ambulance protocols prescribe [31]. Future research should focus on developing tools for scientifically substantiated assistance in this decision-making and improve the quality of pre-hospital triage in severely injured patients [32, 33].

### Strengths and limitations

A strength of this study is that it includes data of all documented severely injured patients over a period of 10 years in one country. There are also some limitations that need to be addressed. We observed an increasing number of (severely) injured patients in the study period. Although we tried to correct for the fact that some NTCs did not participate at the beginning of the DTR, the increase in patient numbers may still, at least partly, be explained by the increasing NTC participation over the years. The increase in trauma patient numbers might also be caused by more accurate registration. Another potential bias was posed by the AIS conversion in 2015, when the way of injury coding in the DTR was changed from the 1998 version of the Abbreviated Injury Scale to the 2005/2008 update version. It is well known that the AIS08 version substantially differs from the AIS98 version with regard to the classification of injury severity and accuracy. Specifically, the AIS08 classification results in less patients being classified as severely injured patients (ISS ≥ 16) and less patients with severe (AIS ≥ 3) neurotrauma. This probably also explains the increase in numbers of patients with minor TBI and the reduction in numbers of severely injured patients with severe neurotrauma, which was on average 66% over the years 2008–2014 and 55% over the years 2015–2018. This assumption is confirmed by Pal et al. who showed an increase in head AIS1 and AIS2 classifications and a decrease in AIS > 3 or higher classifications after using the AIS2008 classification [34]. So, despite our best efforts in reclassifying the AIS98 to AIS08 codes according to Palmer’s model [10], it remains challenging to combine the data of both classifications [10, 34, 35].

Lastly, data on the pre-hospital assumption of injury severity are not available in the National Trauma Registry and could not be obtained from the emergency services due to the strict privacy regulations. Therefore, we could not combine the pre-hospital and National Trauma Registry data to give a better insight into the correlation between the triage and ISS.

## Conclusion

Since the introduction of a well-organized trauma system in the Netherlands, trauma care has become progressively centralized, with more severely injured patients being brought directly to a TC. The injury severity within the group of severely injured patients that are directly transported to a TC has increased slightly in the most recent years, especially in the subgroup of patients with severe neurotrauma. However, still 30% of all severely injured patients is initially brought to a NTC. Future research should focus on improving pre-hospital triage to facilitate swift transfer of the right patient to the right hospital.

## Data Availability

Data is available upon request.
